# Mosaic and Concerted Brain Evolution: The Contribution of Microscopic Comparative Neuroanatomy in Lower Vertebrates

**DOI:** 10.3389/fnana.2019.00086

**Published:** 2019-09-25

**Authors:** Biagio D'Aniello, Anna Di Cosmo, Anna Scandurra, Claudia Pinelli

**Affiliations:** ^1^Department of Biology, University of Naples “Federico II”, MSA Campus, Naples, Italy; ^2^Department of Environmental, Biological and Pharmaceutical Sciences and Technologies, University of Campania “L. Vanvitelli”, Caserta, Italy

**Keywords:** concerted evolution, mosaic evolution, brain evolution, NAPDH-d, FMRFamide

The brain is the most complicated organ, due to which many of its functional and evolutionary processes still remain obscure despite the availability of a vast array of studies. Certain limitations arise from the fact that we are using an organ in order to explain itself and moreover the brain is not complex enough to understand its own complexity. What we do know well is that although all vertebrates share a common basic brain organization, different species exhibit wide variability in terms of volumes and proportional sizes of different subdivisions and neural systems. The variation in the size and composition of the brain often correlates with diverse sensory, behavioral, social, and cognitive skills (Butler and Hodos, 2005). However, the evolutionary mechanisms underlying these functional links are unknown.

Presently, there are two theories attempting to explain the evolutionary patterns observed in the brain of vertebrates. The hypothesis of mosaic brain evolution relies on the selective forces acting on specific areas of the brain, whose adaptive responses do not involve other parts of the brain (Barton and Harvey, 2000). In contrast, the hypothesis of concerted brain evolution assumes that as the regions of the brain are physiologically and developmentally interconnected, any evolutionary change (or a new acquisition) in the brain cannot advance without involving all parts of the brain, thereby leading to a coordinated variation in size (Finlay and Darlington, 1995). However, the factors favoring either of the models of brain evolution are still unclear. It should be emphasized that the brain shows a wide array of functions and interconnections that could have a certain amount of interdependence that is variable between different species. Therefore, concerted or mosaic changes could be related to the degree of functional interdependence; thus, two strictly interconnected brain structures are forced to change together in a coordinated manner, despite the action of selective forces on only one of the two. Hence, the validity of both theories appears to fluctuate between studies, which reduces their general applicability. On the other side, although both the models offer distinct functional hypotheses about the mechanisms of the creation of different neural architectures, they are not mutually exclusive. Indeed, there are numerous examples that indicate a combination of mosaic and concerted evolution within the same species (Gutiérrez-Ibáñez et al., 2014; Herculano-Houzel et al., 2014; Noreikiene et al., 2015; Moore and DeVoogd, 2017).

Most of the studies aimed at understanding evolutionary processes are based on the gross anatomy of the vertebrate brain, which provides evidence supporting both the theories. In mammals, the evolution of the major brain subdivisions supports the concerted brain evolution theory, as changes in their volumes are interconnected (Finlay and Darlington, 1995; Whiting and Barton, 2003; Striedter, 2005). Analogous evidence supporting the concerted brain evolution theory has also been obtained in birds (Gutiérrez-Ibáñez et al., 2014) and cartilaginous fishes (Yopak et al., 2010). On the other hand, the mosaic evolution of the brain has also been recorded in certain parts of the mammalian brain (Barton and Harvey, 2000; Brown, 2001; Dobson and Sherwood, 2011; Hager et al., 2012) and in major subdivisions of the brains of birds (Boire and Baron, 1994; Iwaniuk et al., 2004) and bony fishes (Kotrschal et al., 1998, 2017; Gonzalez-Voyer et al., 2009). Mosaic and concerted brain evolution were also studied in invertebrates. The volumes of optic and antennal regions were positively associated in ants, thus supporting a concerted brain size effect (Gronenberg and Hölldobler, 1999). On the other hand, it appears from studies in wasps that mosaic and concerted brain evolution could not be exclusive alternatives (O'Donnell et al., 2018).

These results, together with the observations that variations in the volume of brain subdivisions in fish are strictly linked to contextual factors, such as habitat and feeding (Kotrschal et al., 1998; Gonzalez-Voyer et al., 2009), social organization (Pollen et al., 2007; Kotrschal et al., 2012), and sexual selection (Gonzalez-Voyer and Kolm, 2010), underline the importance of phenotypic plasticity.

Developmental processes during ontogeny appear also important. For example, indirect support for the mosaic model of brain evolution has been provided by experiments demonstrating differential plasticity in different brain regions in response to environmental conditions experienced during development (Kihslinger and Nevitt, 2006; Gonda et al., 2011, 2012). Furthermore, studies on neurogenesis timing and cell cycle rates during development lead to important insight into evolutionary changes in adult brain region size. An immunohistochemical study based on markers for the cell cycle rates, such as proliferating cell nuclear antigen, phosphorylated histone-3, and bromodeoxyuridine, compared the developing brains of parakeets (*Melopsittacus undulatus*) and quails (*Colinus virginianus*). The results showed that the telencephalic cell cycle rates lengthening significantly later in parakeets than in quail, thus explaining why the telencephalon of adult parakeets occupies a larger part of the entire brain with respect the quails (Charvet and Striedter, 2008). Similar studies in other birds (Charvet and Striedter, 2009, 2010; Charvet et al., 2011) and mammals (Dehay and Kennedy, 2007) drawn the same conclusion. All these studies offer a cellular base for the mosaic brain evolution.

Apart from studies on the relative size of the brain subdivisions, some other contribution to the brain evolution theories also arises from estimates of the phenotypic and genetic correlations between brain regions using quantitative genetic methods. Heritability, evolvability, and genetic correlation on the relative size of the brain and its different regions in the three-spined stickleback (*Gasterosteus aculeatus*) have indicated that different brain regions show a high degree of evolutionary independence due to a low genetic correlation, thus supporting the theory of mosaic evolution (Noreikiene et al., 2015). Likewise, quantitative trait locus mapping studies in mice have demonstrated that brain part size can respond to selection in a largely independent way, as predicted by a mosaic scenario (Hager et al., 2012).

Could studies on the microscopic neuroanatomy in adult samples contribute to the debate on mosaic and concerted brain evolution? To this scope it is possible to rely on immunohistochemical or histochemical studies allowing to compare a suitable number of close species and focus the observations on substances having a large brain distribution, in order to relate different brain areas. Two neurochemicals respond well to these requirements, nicotinamide adenine dinucleotide phosphate–diaphorase (NADPH-d) in the brains of amphibians and FMRFamide (FMRFa)-based immunohistochemistry in bony fishes.

NADPH-d is involved in the production of nitric oxide (NO) from L-arginine through a reaction requiring O_2_ and the mediation of the constitutive neuronal isoform of nitric oxide synthase. NO is one of the most widely distributed brain molecules, due to which NADPH-d-based histochemistry appears extensively distributed in the brain. The brain architecture of NADPH-d was studied in a number of amphibian species belonging to three orders (Pinelli et al., 2014). The latter authors carried out a meta-analysis that integrated all the data related to the distribution of NADPH-d in the brains of amphibians and performed a cluster analysis based on the degree of dissimilarities between species. The results showed that the complete brain dendrogram obtained by hierarchical clustering was similar to that achieved using morphological data (Ford and Cannatella, 1993) and molecular biological studies on amphibians based on mitochondrial and nuclear DNA (Roelants and Bossuyt, 2005; Zhang et al., 2005; Pyron and Wiens, 2011), thus revealing the evolutionary history of amphibians. However, when the same analysis was applied to the major brain subdivisions, only the hindbrain was in line with the phylogenetic tree of amphibians, whereas the other brain areas showed similarities between distant species. For example, the apodan *Dermophis mexicanus* had a more similar diencephalic NADPH-d pattern to the anurans than the urodele *Pleurodeles waltl*. A similar conclusion can be drawn after comparing the other brain subdivisions with the hindbrain. In general, it appears that the NADPH-d system in the forebrain of amphibians is more variable than in the hindbrain, implying that convergent evolutionary patterns based on specific adaptations occur in the forebrain without involving the hindbrain. In a concerted evolution, it is often expected that any evolutionary change in an area of the brain will involve other brain areas, thus always giving the same evolutionary dendrogram when performing a hierarchical clustering. However, this does not actually happen. The mosaic evolution better explains these results, owing to the fact that the selective forces specifically act on certain brain areas without involving the others.

Similar reasoning can be achieved while analyzing studies describing the immunohistochemical distribution of FMRFa in the fish brain. It is well-known that the brains of fish show great morphological variability due to the high number of ecological, behavioral, and social processes that they are subjected (Kotrschal et al., 1998; Pollen et al., 2007; Lecchini et al., 2014). Accordingly, different natural populations of the same species can show variation in the relative brain size in response to the habitat (Gonda et al., 2011). Among the most variable brain areas are the olfactory bulbs (OBs), which, according to the model of mosaic evolution, show positive bivariate allometry when compared with the other brain areas (Gonzalez-Voyer et al., 2009). The OBs of teleostean can have three different neuroanatomical relationships with the telencephalon. The type I morphology relies on sessile conditions in which the OBs are merged with the ventral telencephalic area (VTA). In the type II morphology, OBs are closely connected to the VTA through a short peduncle in a pseudo-sessile condition. In species with type III morphology, OBs are stalked and connected with the VTA through a long peduncle (D'Aniello et al., 2016). Based on the number and size of FMRFa immunoreactive (FMRFa-ir) cells and the topology of the OBs, the authors obtained a dendrogram based on the similarities between the studied species. The results showed several deviations from the formal teleost phylogenetic tree (Betancur-R et al., 2013), thus supporting the theory that the adaptive plasticity largely overlaps evolutionary constraints in the olfactory system, allowing phenomena of divergence or convergence among close species (Gonda et al., 2011, 2012; Eifert et al., 2015). However, looking at the brain areas that are unrelated to the olfactory system, the FMRFa-ir system in fish appears quite constant irrespective of the topology of the OBs. Indeed, FMRFa-ir cells are similarly arranged in the diencephalon, with a distribution in the dorsal and ventral hypothalamus and a comparable cell size (Bonn and König, 1989; Batten et al., 1990; Ostholm et al., 1990; Fujii and Kobayashi, 1992; Rama and Subhedar, 1992; Pestarino and Vallarino, 1996; Pinelli et al., 2000; unpublished data from D'Aniello et al., 2016). Thus, FMRFa-ir neuronal changes in the olfactory system do not involve central FMRFa-ir neurons, again supporting the mosaic brain evolution theory.

The distribution of neuromarkers in the adult brains and during development are only a few examples demonstrating that studies on the microscopic neuroanatomy are highly useful in evaluating evolutionary theories giving support to mosaic brain evolution. In our opinion, similar studies could provide important insights to identify trends in brain evolution and should be considered and implemented very carefully.

## Author Contributions

BD'A, AD, AS, and CP have made a substantial, direct and intellectual contribution to the work, and approved it for publication.

### Conflict of Interest

The authors declare that the research was conducted in the absence of any commercial or financial relationships that could be construed as a potential conflict of interest.
